# A pre-protective strategy for precise tumor targeting and efficient photodynamic therapy with a switchable DNA/upconversion nanocomposite†
†Electronic supplementary information (ESI) available. See DOI: 10.1039/c8sc00098k


**DOI:** 10.1039/c8sc00098k

**Published:** 2018-03-05

**Authors:** Zhengze Yu, Yegang Ge, Qiaoqiao Sun, Wei Pan, Xiuyan Wan, Na Li, Bo Tang

**Affiliations:** a College of Chemistry, Chemical Engineering and Materials Science , Collaborative Innovation Center of Functionalized Probes for Chemical Imaging in Universities of Shandong , Key Laboratory of Molecular and Nano Probes , Ministry of Education , Institute of Molecular and Nano Science , Shandong Normal University , Jinan 250014 , P. R. China . Email: lina@sdnu.edu.cn ; Email: tangb@sdnu.edu.cn

## Abstract

Tumor-specific targeting based on folic acid (FA) is one of the most common and significant approaches in cancer therapy. However, the expression of folate receptors (FRs) in normal tissues will lead to unexpected targeting and unsatisfactory therapeutic effect. To address this issue, we develop a pre-protective strategy for precise tumor targeting and efficient photodynamic therapy (PDT) using a switchable DNA/upconversion nanocomposite, which can be triggered in the acidic tumor microenvironment. The DNA/upconversion nanocomposite is composed of polyacrylic acid (PAA) coated upconversion nanoparticles (UCNPs), the surface of which is modified using FA and chlorin e6 (Ce6) functionalized DNA sequences with different lengths. Initially, FA on the shorter DNA was protected by a longer DNA to prevent the bonding to FRs on normal cells. Once reaching the acidic tumor microenvironment, C base-rich longer DNA forms a C-quadruplex, resulting in the exposure of the FA groups and the bonding of FA and FRs on cancer cell membranes to achieve precise targeting. Simultaneously, the photosensitizer chlorin e6 (Ce6) gets close to the surface of UCNPs, enabling the excitation of Ce6 to generate singlet oxygen (^1^O_2_) under near infrared light *via* Förster resonance energy transfer (FRET). *In vivo* experiments indicated that higher tumor targeting efficiency was achieved and the tumor growth was greatly inhibited through the pre-protective strategy.

## Abstract 2

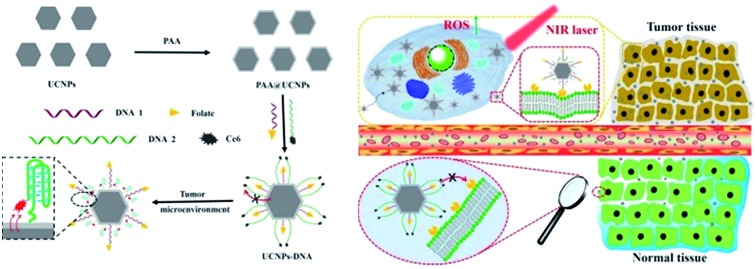
A pre-protective strategy for precise tumor targeting and efficient photodynamic therapy was developed using a switchable DNA/upconversion nanocomposite.

## Introduction

Cancer has become a major killer that threatens human life and health because of the rapid proliferation and metastasis of cancer cells.^1^,^2^ With the deeper understanding of cancer in the past decades, targeted cancer therapy has been one of the main choices in clinical trials.^3^–^6^ Based on the overexpression of the receptors on cancer cell membranes, employing corresponding ligands to improve the targeting efficiency was the most common method.^7^–^9^ By conjugating a series of targeting groups (*e.g.* FA, TAT peptide, c(RGDyC) peptide, and aptamers) on the nanoparticles, tumor-specific targeting can be realized.^10^–^14^ Among these approaches, the FA group has been widely used for tumor targeting due to its easy modification and good targeting efficiency.^15^–^17^ However, there is a key issue that has always been ignored, that is, folate receptors are not only overexpressed at high levels on the membrane of nonmalignant proliferative cells (*e.g.* bone marrow cells and follicle cells), but also expressed at low levels in most normal tissues, which tremendously reduces the targeting efficiency and therapeutic effect due to the large population of normal cells.^18^–^20^ Therefore, an effective strategy that can protect the targeting groups and decrease unexpected bonding is urgently needed.

As an important treatment for cancer, PDT possesses superior advantages over other therapeutic methods due to its minimal invasiveness, fast healing process and potential repeatability.^21^–^27^ A photosensitizer is indispensable for the application of PDT in cancer therapy, however, traditional photosensitizers lack active targeting ability. When the photosensitizer circulates in the body, the retention of agents in the tumor tissues is limited. In this case, the therapeutic effect of PDT without tumor targeting will inevitably be greatly reduced.^28^,^29^ Besides, the poor penetration of ultraviolet or visible excitation light in conventional PDT also severely limits its clinical application. Improving the penetration of excitation light is also a key factor for enhancing the treatment effects of PDT.^30^–^34^ Thus, precise tumor targeting accompanied by NIR excitation would be extremely favorable to improve the therapeutic effect of PDT.

**Scheme 1:**
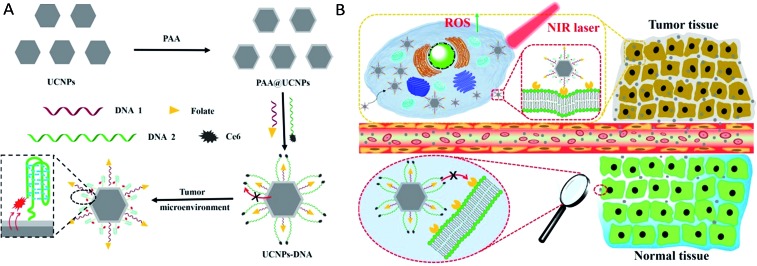
(A) Synthesis process of UCNPs@PAA–DNA. (B) The details of precise tumor targeting and specific PDT for cancer.

Herein, we present a pre-protective strategy for precise tumor targeting and enhanced PDT by constructing a dynamic DNA/UCNP nanocomposite. Two kinds of DNA sequences with different lengths and modifications are anchored on the surface of polyacrylic acid (PAA) coated UCNPs. In normal tissues, FA groups on the shorter DNA sequences are first covered and protected by the longer DNA sequences to separate from FRs, which could avoid unexpected uptake. Once the nanocomposites reach the tumor region, C base-rich longer DNA would fold in the acidic tumor microenvironment and shorten.^35^,^36^ Thus, FA groups were exposed and further bond to FRs on the membrane of cancer cells, thereby achieving the precise targeting of nanocomposites. At the same time, the photosensitizer Ce6 on the longer DNA gets close to the surface of UCNPs, and can be excited to generate ^1^O_2_ under NIR *via* FRET due to the fluorescence spectra match,^37^–^42^ which can be employed to destroy tumor cells for cancer therapy. The construction of the nanocomposite (UCNPs@PAA–DNA) and details of precise tumor targeting and specific PDT are illustrated in Scheme 1.

## Results and discussion

### Synthesis and characterization of the nanocomposite

**Fig. 1:**
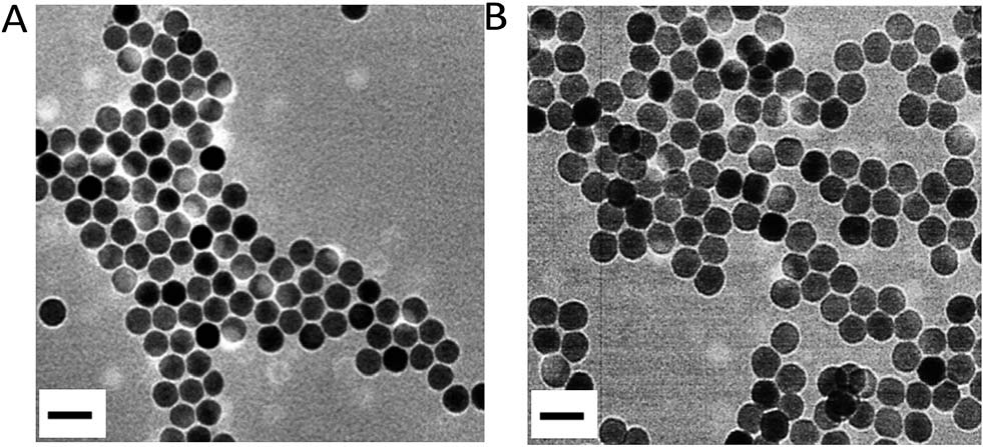
HRTEM images of OA–UCNPs in cyclohexane (A) and UCNPs@PAA in the aqueous phase (B). Scale bars are 50 nm.

**Fig. 2:**
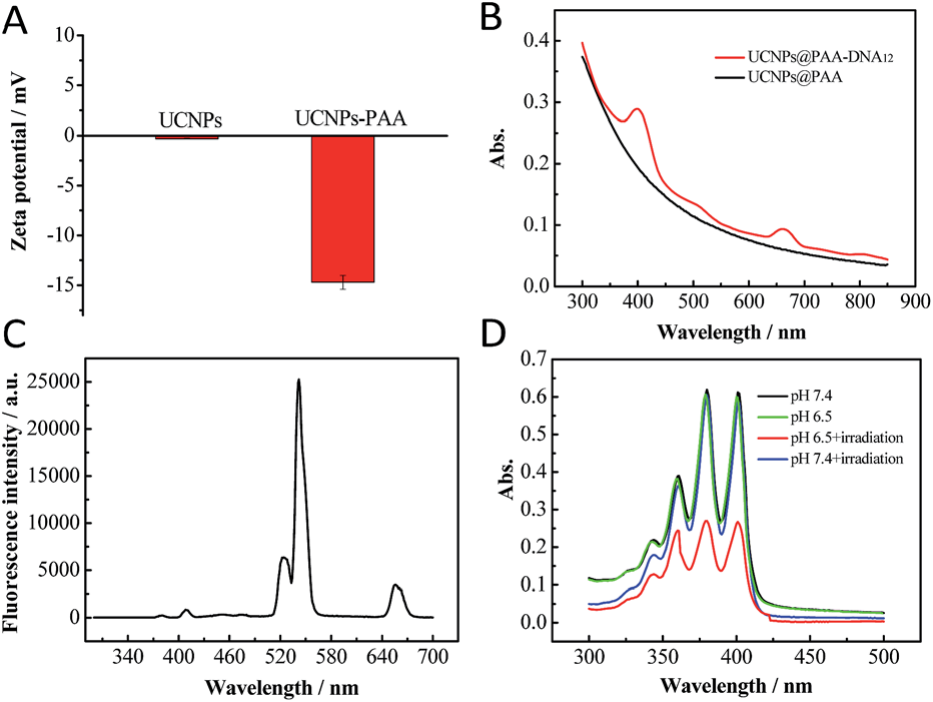
(A) Zeta potential of UCNPs and UCNPs@PAA. (B) Absorption spectra of UCNPs@PAA and UCNPs@PAA–DNA_1/2_. (C) Fluorescence spectrum of UCNPs@PAA. The characteristic emission peaks appeared at 521 nm, 541 nm and 655 nm due to the Er^3+^ doping. (D) Absorption spectra of ABMD with UCNPs@PAA–DNA_1/2_ under different conditions: pH 7.4 without irradiation, pH 6.5 without irradiation, pH 7.4 with irradiation and pH 6.5 with irradiation. The laser was operated at a power of 1.5 W cm^–2^ for 5 min.

β-Phase UCNPs NaYF_4_ doped with 20% Yb^3+^ and 0.2% Er^3+^ (NaYF_4_:Yb^3+^,Er^3+^) were first prepared through an established solvothermal method according to a previous study with some modifications.^43^,^44^ These as-synthesized oleic acid (OA)-conjugated UCNPs were then coated with polyacrylic acid (PAA) to improve the water solubility and biocompatibility, and to facilitate subsequent DNA conjugation as well.^45^ As shown in the high resolution transmission electron microscopy (HRTEM) images in Fig. 1, OA–UCNPs exhibit a uniform hexagonal morphology with a diameter of about 25 nm, and disperse well in cyclohexane. After PAA coating, UCNPs@PAA possesses good monodispersity in water and the size of the nanoparticles increases to 35 nm. The zeta potentials change from –0.32 ± 0.04 mV to –14.7 ± 0.75 mV due to the introduced carboxyl groups of PAA on the surface of the nanoparticles (Fig. 2A). Finally, FA and Ce6 conjugated DNA sequences with different lengths (Table S1†) at a particular ratio were anchored on the nanoparticles *via* amido bonds, which further improves the biocompatibility after DNA decoration.^46^ From the absorption spectra in Fig. 2B, obvious absorption peaks of Ce6 at around 400 nm and 655 nm appear after DNA modifications, which provides further evidence for the successful fabrication of UCNPs@PAA–DNA.

### Acid triggered ^1^O_2_ generation

Acid environment triggered C-rich DNA folding was verified. A C-rich DNA sequence modified with Cy3 and BHQ1 was designed to demonstrate the C-quadruplex i-motif structure formation of C-rich DNA at pH 6.5 through fluorescence analysis (DNA4, Table S1†). As shown in Fig. S1 in the ESI,† the fluorescence intensity of Cy3 decreased obviously when DNA4 was at pH 6.5, which indicated that Cy3 was quenched by BHQ1 and further confirmed the DNA folding. Then we verified whether the nanocomposite could generate ^1^O_2_ in the mild acid environment under NIR irradiation. As shown in Fig. 2C, there are three characteristic emission peaks in the fluorescence spectrum of the UCNPs NaYF_4_:Yb^3+^,Er^3+^ at 521 nm, 541 nm and 655 nm, which corresponded to ^2^H_11/2_–^4^H_15/2_, ^4^S_3/2_–^4^S_15/2_, and ^4^F_19/2_–^4^I_15/2_ transformations of Er^3+^, respectively.^47^,^48^ Combined with the absorption spectrum of UCNPs@PAA–DNA_1/2_, we found that the absorption band of Ce6 around 655 nm overlapped well with the UCNP emission, which meets the needs of FRET. A special probe, 9,10-anthracenediyl-bis(methylene)dimalonic acid (ABMD), was employed for the detection of ^1^O_2_. As shown in Fig. 2D, the sharp decrease in the absorption of ABMD provided compelling evidence for the ^1^O_2_ generation when UCNPs@PAA–DNA_2_ was irradiated with 980 NIR light at pH 6.5, which was due to the acid environment triggering the C-quadruplex i-motif structure formation and FRET occurrence. The kinetic study showed that ^1^O_2_ was produced continuously with increasing irradiation time (Fig. S2, ESI†). As a control, when the pH was 7.4, there was no change in the absorption spectrum, which revealed no ^1^O_2_ generation and no side effects in the neutral normal tissues. This is because the C-rich longer DNA still maintains the original state under neutral conditions so that Ce6 molecules remain far away from the UCNP surface. These above results indicated that the acid environment could trigger the structure switch of the C base-rich DNA sequence and the nanocomposite could then generate ^1^O_2_ under NIR irradiation.

### Optimization of the DNA ratio

**Fig. 3:**
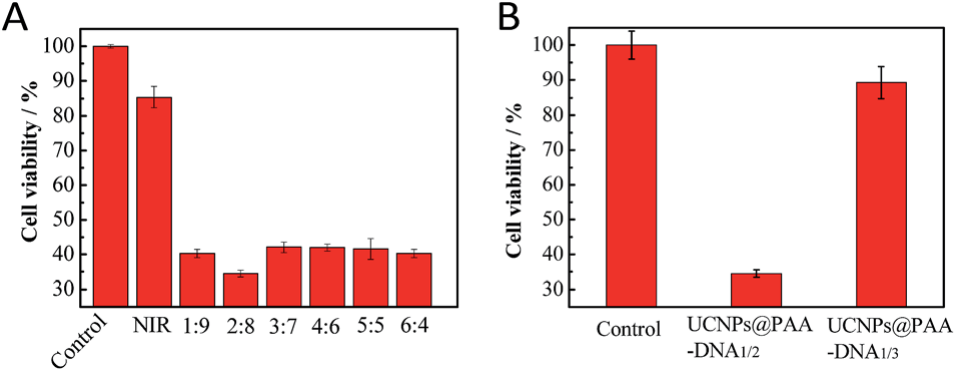
MTT assays. The cell viability when incubated with UCNPs@PAA–DNA_1/2_ with different ratios of DNA1 and DNA2 (A), UCNPs@PAA–DNA_1/2_ and UCNPs@PAA–DNA_1/3_ (B) at pH 6.5 under 5 min irradiation.

To maximize the therapeutic effect, the ratio of shorter and longer DNAs per nanocomposite was optimized. Considering that more FA conjugated DNA1 would allow more cellular uptake and more Ce6 conjugated DNA2 would result in more ^1^O_2_ generation, the ratio of these two kinds of DNAs was of great importance. The total amount of DNAs on the nanocomposite surface was determined using fluorescence quantitative analysis. Different amounts of longer DNA2 first reacted with a certain amount of UCNPs@PAA for 12 h, and then the fluorescence of Ce6 in the supernatant was measured. As shown in Fig. S3 in the ESI,† when the amount of longer DNA2 reached 1.7 nmol, an obvious emission peak appeared in the fluorescence spectra, which indicated that the threshold amount of total DNA was 1.5 nmol. Subsequently, nanocomposites with various shorter and longer DNA ratios were prepared. The optimal ratio was based on the viability of cancer cells. We chose human breast cancer cells (MCF-7) as a cell model due to their high FR level (Fig. S4, ESI†). After incubation at pH 6.5 for 12 h, human breast cancer cells (MCF-7) were irradiated with 980 nm NIR for 5 min. Then we conducted MTT (3-(4,5-dimethylthiazol-2-yl)-2,5-diphenyltetrazolium bromide) assays to evaluate their ability in inducing cell apoptosis^49^,^50^ and further made the optimal choice on the DNA ratio. As shown in Fig. 3A, the nanocomposite with the DNA ratio of 2 : 8 treated MCF-7 cells exhibited the minimum value in viability, demonstrating that this DNA ratio was the best. Thus, the nanocomposite with the DNA ratio of 2 : 8 was chosen for further use. What's more, to demonstrate the precise targeting and therapeutic ability of our designed nanocomposite, we constructed UCNPs@PAA–DNA_1/3_ as a negative control, in which C base-rich DNA2 was replaced by a scrambled sequence DNA3. As DNA3 could not switch its structure in an acid environment, FA was protected all the time and Ce6 remained far away from the UCNP surface, and both the targeting and therapeutic functions were restricted. After incubation with UCNPs@PAA–DNA_1/3_, most of the cells were still alive and displayed high viability up to about 90%. However, the viability of cancer cells incubated with UCNPs@PAA–DNA_1/2_ at pH 6.5 decreased markedly, suggesting excellent therapeutic effects (Fig. 3B).

### FA mediated cellular uptake

**Fig. 4:**
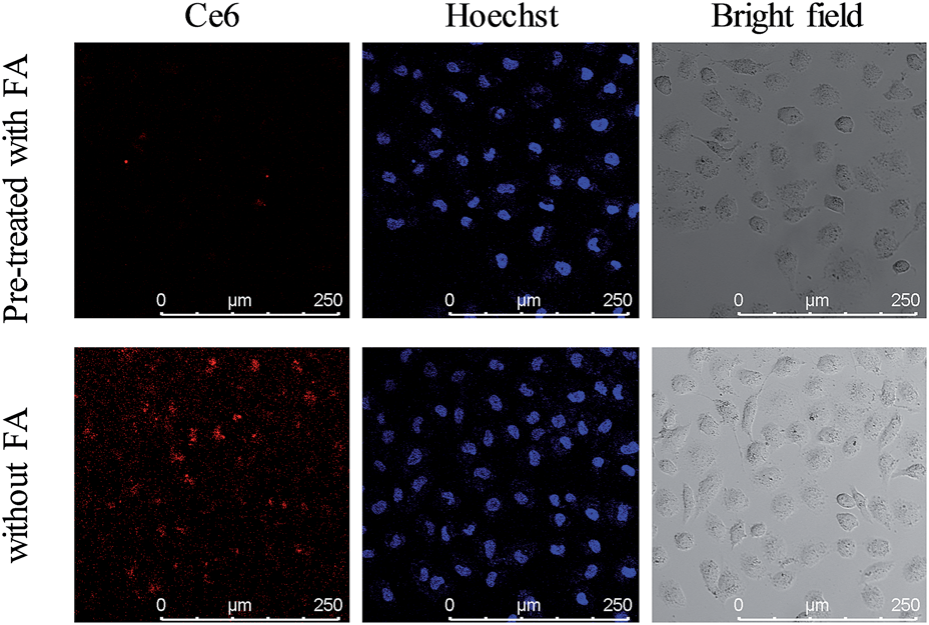
Confocal images of MCF-7 cells incubated with UCNPs@PAA–DNA_1/2_ at pH 6.5 after cells were pre-treated with FA (top) or not (bottom).

FRs are generally overexpressed on the membrane of cancer cells and are expressed at low levels on normal cells. The presence of FA on the nanoparticles leads to the feature of specific recognition. The FR mediated uptake of the nanocomposite was investigated by employing a confocal laser scanning microscope (CLSM). As shown in Fig. 4, a bright red fluorescence signal of Ce6 was observed in the MCF-7 cells incubated with the UCNPs@PAA–DNA_1/2_. However, it was invisible when MCF-7 cells were pre-treated with FA before incubation with the nanocomposite, which was due to the competitive manner of free FA molecules.^51^ And a similar trend was observed in the fluorescence quantification analysis (Fig. S5, ESI†). Imaging flow cytometry (IFC) experiments were also carried out to confirm the FR mediated cell uptake. As shown by confocal images and statistical data, cells without pre-treated FA molecules exhibited stronger fluorescence intensity than FA treated cells (Fig. S6, Table S2, ESI†). The results verified that the nanocomposite is internalized *via* FRs. As reported in previous studies,^52^ FA conjugated nanoparticles would be finally located in lysosomes when they are internalized *via* FRs, which is beneficial for C-quadruplex i-motif structure formation and ^1^O_2_ generation.

### *In vitro* targeting experiment

**Fig. 5:**
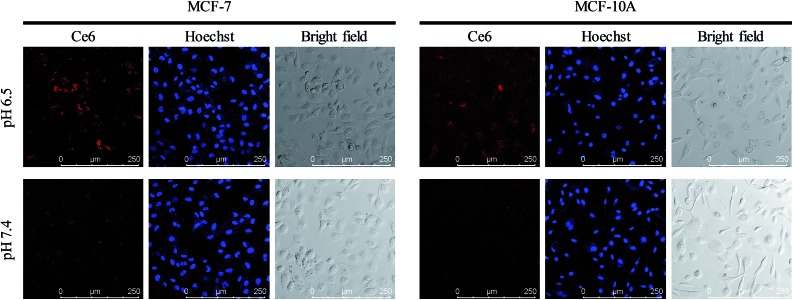
Precise targeting experiments. Confocal images of MCF-7 cells and MCF-10A cells incubated with UCNPs@PAA–DNA_1/2_ at pH 6.5 and 7.4.

Acid microenvironment triggered specific targeting of cancer cells was then verified in MCF-7 cells using CLSM. As shown in the confocal images in Fig. 5, there was a bright red signal when MCF-7 cells were treated with the nanocomposite at pH 6.5, which resulted from the exposure of FA to the FRs on the cancer cell membrane. However, as neutral pH would not trigger the structural change of longer DNAs and the FA groups on the shorter DNAs were still protected, the signal was very weak when the incubation pH was 7.4. In addition, due to the low expression level of FRs on the membrane of normal human breast cells (MCF-10A), the cells exhibited a weak signal of Ce6 both at pH 6.5 and 7.4. Interestingly, the signal from MCF-10A cells at pH 6.5 was a little stronger than that at pH 7.4, indicating that the FA conjugated nanoparticles could target normal cells and may cause side effects if the FA remained exposed without protection. And the same results in fluorescence quantification further confirmed the ability of specific targeting of cancer cells and avoiding entrance into normal cells (Fig. S7, ESI†). IFC images and corresponding statistical data also evidenced the acid triggered specific targeting of cancer cells by the nanocomposite (Fig. S8, ESI†).

### *In vivo* targeting experiment

**Fig. 6:**
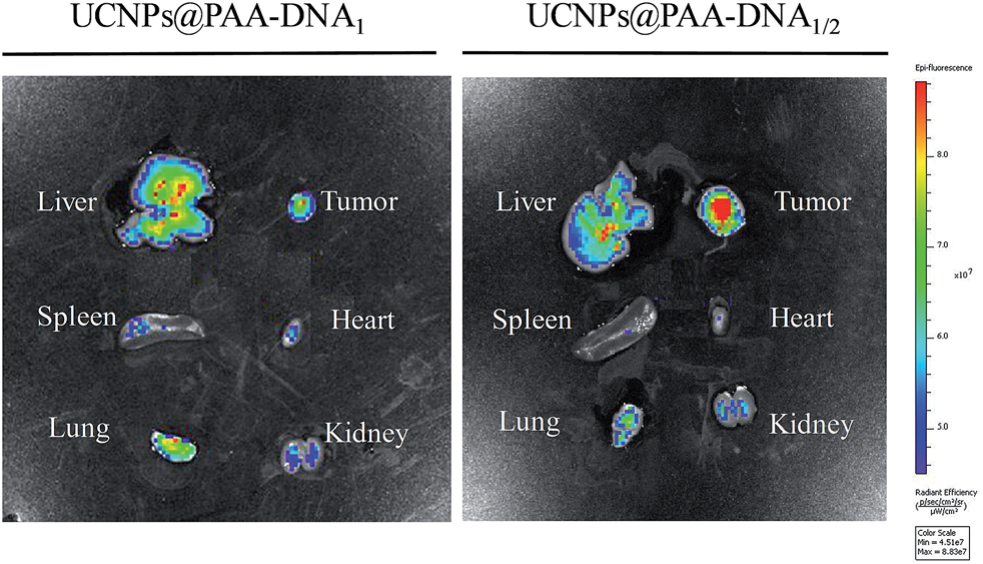
*In vivo* precise targeting. *In vivo* imaging of five major organs harvested from a mouse at 8 h post-injection with UCNPs@PAA–DNA_1_(Ce6) (left) or UCNPs@PAA–DNA_1/2_ (right).

As the overexpression of FRs on the membrane of normal cells would sharply reduce the targeting efficiency and cause unexpected side effects, precise targeting of the tumor tissue by FA conjugated nanoparticles is of great significance for cancer therapy. To demonstrate the superior targeting ability of the nanocomposite, we conducted *in vivo* tumor targeting experiments in Balb/c mice with xenograft tumors. The mice were injected with the nanocomposite (4 mg kg^–1^) *via* intravenous administration. To highlight the superiorities of our designed nanocomposite as a precise targeting therapeutic agent, we chose UCNPs@PAA–DNA_1_(Ce6) as the control by modifying equal amount of Ce6 on the surface of UCNPs@PAA–DNA_1_, which represented the traditional FA conjugated nanoparticles. As shown in Fig. 6, besides the tumor region, UCNPs@PAA–DNA_1_(Ce6) are distributed in all the major organs (heart, liver, spleen, lungs, and kidneys), and only a small percentage of the injection dose reached the tumor tissue. However, as for UCNPs@PAA–DNA_1/2_, the fluorescence signal in tumor tissues was greatly enhanced, and the amount of nanocomposite in five major organs decreased sharply at the same time. The above results indicated that FRs on the membrane of normal cells would indeed interfere with the targeting effect of the traditional FA conjugated nanoparticles on the tumor tissue and our designed nanocomposite could achieve precise tumor targeting and reduced side effects on normal tissues. The targeting effect of UCNPs@PAA–DNA_1/2_ on tumors was quantified to be 12.1 ± 0.7% ID per g using inductively coupled plasma-atomic emission spectrometry (ICP-AES), which is higher than that of UCNPs@PAA–DNA_1_(Ce6) (4.1 ± 0.4% ID per g) (Fig. S9, ESI†). The metabolism kinetic study of UCNPs@PAA–DNA_1/2_ was also carried out by collecting the feces and urine. As shown in Fig. S10 in the ESI,† the retention content of nanoparticles in the body decreased to less than 5% at 72 h post injection. And ^1^O_2_ generation of UCNPs@PAA–DNA_1/2_ at pH 6.5 with human serum albumin (HSA, 10 mg mL^–1^) was detected by ABMD. As shown in Fig. S11 in the ESI,† the absorption of ABMD decreased obviously and was similar to that without HSA, which indicated that the protein has a negligible influence on our designed nanoparticles.

### Tumor growth inhibition *in vivo*

**Fig. 7:**
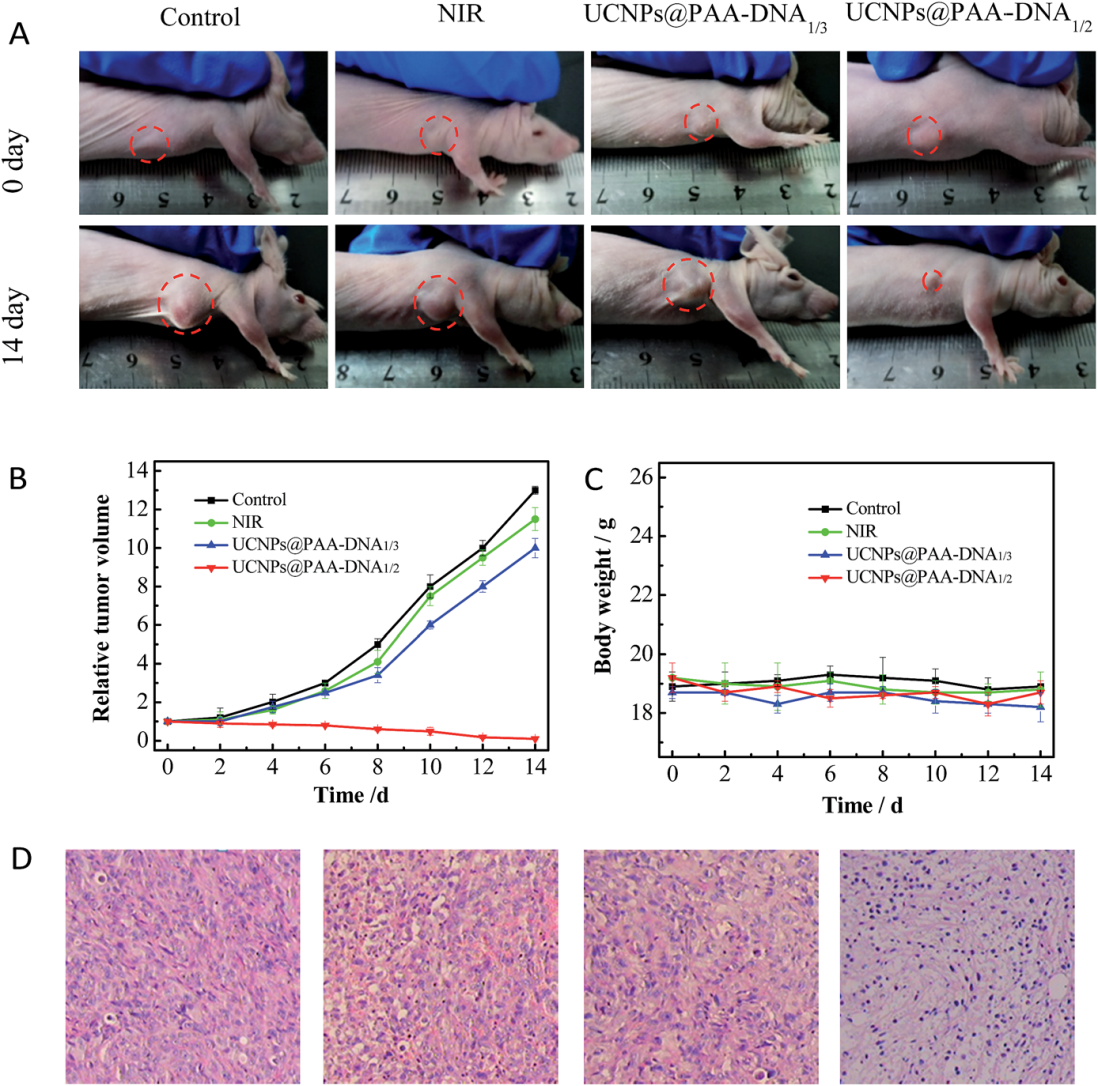
*In vivo* therapeutic effects. (A) Photographs of mice with different treatments on day 0 (top) and day 14 (bottom). The laser was operated at a power of 1.5 W cm^–2^ for 5 min. Tumor growth curves (B) and mice body weight curves (C) of different groups of tumor-bearing mice. (D) H&E staining of tumors in mice that received different treatments.

Encouraged by the outstanding therapeutic effect *in vitro* and good targeting ability *in vivo*, we then evaluated the efficacy in tumor growth inhibition. Nude mice were subcutaneously injected with MCF-7 cells to form a xenograft tumor. When the solid tumor reached about 150 mm^3^, the mice received different treatments: PBS, NIR irradiation only, UCNPs@PAA–DNA_1/3_ with NIR irradiation, and UCNPs@PAA–DNA_1/2_ with NIR irradiation. The nanoparticles were injected *via* intravenous administration only once and NIR irradiation was conducted for 5 min (1.5 W cm^–2^) at 8 h post injection. The tumor volume was monitored every other day in a period of 14 days without extra irradiation. As shown in Fig. 7, the volume of tumors in mice treated with PBS or NIR only increased greatly and was about 12 fold larger than its initial volume. As DNA3 could not change its structure in an acid environment, FA groups could be protected and Ce6 could not get close to the surface of UCNPs, resulting in low targeting efficiency and little ^1^O_2_ generation. Similarly, the tumor volume was found to increase nearly 10-fold over this period. Remarkably, the tumor was almost eliminated after being treated with our designed nanocomposite, due to the acid microenvironment triggered precise targeting, which demonstrated its excellent therapeutic effect (Fig. 7A and B). In addition, H&E staining on tumor sections provided further evidence for the treatment efficacy of inducing tumor cell apoptosis. As shown in H&E staining images, UCNPs@PAA–DNA_1/2_ treated tumors exhibited extensive apoptotic cells, which was reflected in a large amount of nuclear shrinkage and fragmentation (Fig. 7D). Meanwhile, no obvious body weight change was observed during the treatments, suggesting that the nanocomposite would not induce negative system toxicity (Fig. 7C). H&E staining on five major organs was also conducted at the end of the treatment period and no histopathological abnormalities appeared in all organs of mice with different treatments (Fig. S12, ESI†).

## Conclusions

In summary, a pre-protective strategy was introduced for precise tumor targeting and efficient PDT with a switchable DNA/upconversion nanocomposite. The nanocomposite consists of PAA coated UCNPs that are conjugated with FA and Ce6 functionalized DNA sequences. In normal tissues, the FA group of the shorter DNA sequence was protected by the longer DNA sequence to prevent the conjugation to FRs. Once reaching the tumor region, C base-rich longer DNA would form C-quadruplex and FA groups will be exposed to bond FRs on the membrane of cancer cells, enabling precise tumor targeting. Simultaneously, the photosensitizer Ce6 on the longer DNA moved exactly close to the surface of UCNPs and could be activated to produce ^1^O_2_ under NIR *via* FRET. *In vivo* experiments demonstrated that nanocomposites accumulated in the tumor region and the tumor growth was severely inhibited, suggesting that the targeting efficiency and therapeutic effect of PDT were greatly improved. We anticipate that this pre-protective strategy could provide new insights for precise targeting and highly efficient cancer therapy.

## Conflicts of interest

The authors declare no competing financial interest.

## Supplementary Material

Supplementary information
