# Supplementary material for: A pre-protective strategy for precise tumor targeting and efficient photodynamic therapy with a switchable DNA/upconversion nanocomposite
Source: Chem Sci. 2018 Mar 5;9(14):3563–9. doi: 10.1039/c8sc00098k (PMC5934823; doi:10.1039/c8sc00098k)
Supplement: Supplementary file 1 [file SC-009-C8SC00098K-s001.pdf]

Electronic Supplementary Material (ESI) for Chemical Science.
This journal is © The Royal Society of Chemistry 2018

Supplementary information

A pre-protective strategy for precise tumor targeting and efficient photodynamic therapy with a switchable DNA/upconversion nanocomposite

Zhengze Yu, Yegang Ge, Qiaoqiao Sun, Wei Pan, Xiuyan Wan, Na Li,* and Bo Tang*

College of Chemistry, Chemical Engineering and Materials Science, Collaborative Innovation Center of Functionalized Probes for Chemical Imaging in Universities of Shandong, Key Laboratory of Molecular and Nano Probes, Ministry of Education, Institute of Molecular and Nano Science, Shandong Normal University, Jinan 250014, P. R. China.

E-mail: lina@sdnu.edu.cn, tangb@sdnu.edu.cn

Materials.

Ethyl alcohol (CH_3CH_2OH), methyl alcohol (CH_3OH), cyclohexane and concentrated hydrochloric acid were purchased from China National Pharmaceutical Group Corporation (Shanghai, China). 1-octadecene (ODE), rare earth oxides yttrium(III) oxide (Y_2O_3), ytterbium(III) oxide (Yb_2O_3), thulium(III) oxide (Er_2O_3) and 3-(4,5-dimethyl- thiazol-2-yl)-2,5-diphenyltetrazolium bromide (MTT) were purchased from Sigma Chemical Company; oleic acid (OA), 1-ethyl-3-(3-(dimethylamino)propyl) carbodiimide hydrochloride (EDC) and N-hydroxysuccinimide (NHS) were purchased from Alfa Aesar Chemical Ltd. (tianjin, China); The DNA of folic acid and Ce6 is produced by the TakaRa company in Dalian. Oleic acid and 1-octadecene (ODE) were of technical grade and the others were of analytical grade. All the chemicals were used without further purification. The human

breast cancer cell line (MCF-7) and normal breast cell line (MCF-10A) was purchased from KeyGEN biotechnology Company (Nanjing, China).

Instruments.

High resolution transmission electron microscopy (HRTEM) was taken on a JEM-2100 electron microscope. Fluorescence spectra were acquired from FLS-980 Edinburgh Fluorescence Spectrometer with a Xenon lamp. Absorption spectra were carried out on a pharmaspec UV-1700 UV-visible spectrophotometer (Shimadzu, Japan). Absorbance was measured in a microplate reader (Synergy 2, Biotek, USA) in the MTT assay. Success of each reaction step was confirmed by monitoring the changes in zeta potential with a Malvern Zeta Sizer Nano (Malvern Instruments). A TCS SP5 confocal laser scanning microscopy (Leica Co., Ltd. Germany) was used for performing the confocal fluorescence imaging.

Synthesis of $NaYF_4$:Yb/Er (20/0.2) upconversion nanocrystals. The NaYF4:Yb^{3+}, Er^{3+} nanocrystals were prepared via a solvothermal method with some modifications. To obtain rare earth chlorides, 1 mmol rare earth oxides Y_2O_3, Yb_2O_3, and Er_2O_3 with a stoichiometric ratio of 79.8:20:0.2 were dissolved in hydrochloric acid, and then the solution was stirred and heated to evaporate the water completely. In the typical synthesis procedure, YCl_3 (0.798 mmol), $YbCl_3$ (0.20 mmol), and $ErCl_3$ (0.002 mmol) were dispersed in oleic acid (OA, 8 mL) and 1-octadecene (ODE, 18 mL), then the mixture was heated to 160 ^oC for 30 min. After a homogeneous solution was formed, the mixture was cooled to room temperature. Then, NaOH (0.1 g, 2.5 mmol) and NH_4F (0.148 g, 4 mmol) in 10 mL of methanol solution were added dropwise under

vigorously stirring for 30 min. The temperature was heated to 100 ^{o}C to evaporate methanol, then was raised to 295 ^{o}C in an argon atmosphere for 90 min and finally cooled down to room temperature naturally. The resulting $NaYF_4$:Yb,Er nanoparticles were precipitated by adding ethanol and then centrifuged and washed with ethanol and cyclohexane for several times. The precipitates were redispersed in 10 mL hexamethylene solution.

Synthesis of PAA@UCNPs. The PAA@UCNPs synthesis was approved by a literature protocol reported previously.[43] Briefly, 150 mg of PAA (1800 Da) and 15 mL DEG were added to a three-neck flask. The mixture was heated to 110 °C to procedure a cleared solution. Then hexamethylene solution containing 20 mg UCNPs was added slowly and the solution was maintained at 110 °C for 1 h under nitrogen protection. After heating at 240 °C for 1.5 h, the resultant solution was cooled down to room temperature and precipitated with ethanol. The obtained PAA@UCNPs was recovered by centrifugation (14000 rpm for 10 min) and washed three times with ethanol/water (1:1 v/v).

Synthesis of DNA linking of upconversion nanoparticles (UCNPs@PAA-DNA). The PAA-capped UCNPs were covalently encapsulated with DNA as reported previously. Briefly, 0.5 mg of PAA@UCNPs in 1 mL MES buffer (10 mM, pH 6.0) were activated by EDC and NHS (680 nM) to form the active succinimidyl ester for 30 min. Then the DNA that was dissolves in water was added to the mixture. After 24 h magnetic stirring in dark, the UCNPs@PAA-DNA was purified by centrifugation (14000 rpm for 10 min) and washed with water to remove excess reactant.

***In vitro* detection of $^1O_2$.** UCNPs@PAA-$DNA_{1/2}$ (100 μg/mL) was dispersed in PBS buffer with different pH (6.5 or 7.4) containing a special probe 9,10-anthracenediyl-bis(methylene)dimalonic acid (ABMD, 0.1 mM). Then the solution was conducted with laser irradiation (1.5 W·cm^{-2}, 5 min) or not. The solution was then centrifuged and the supernatant was measured to obtain UV-vis spectra.

Cell culture. MCF-7 and MCF-10a cells were cultured in Dulbecco's modified Eagles medium (DMEM) with 10% fetal bovine serum and 100 U/ml 1% antibiotics penicillin/streptomycin and maintained at 37 °C in a 100% humidified atmosphere containing 5% CO_2.

***In vitro* therapeutic effects and the optimization of DNA proportions.** Firstly, UCNPs@PAA-$DNA_{1/2}$ with different DNA ratio was synthesized. The proportions 1:9, 2:8, 3:7, 4:6, 5:5, 6:4 (DNA1:DNA2) were chosen. Then MCF-7 cells were incubated in 96-well microliter plates for 24 h. After that the different proportions of UCNPs@PAA-$DNA_{1/2}$ (0.1 mg/mL) in DMEM culture medium was added to the cells. After 12 h, the cells was washed with PBS buffer to remove the NPs that were not ingested into the cells. Then, the cells were treated with irradiation of laser powers (1.5 W·cm^{-2}) for 5 min. The control group was without any treatment. Then, the cells were further incubated for 24 h. Next, 150 μL MTT solution (0.5 mg/mL) was added to each well to cultivate for 4 h. After removing the MTT solution, 150 μL of DMSO was injected to each well. The absorbance was measured at 490 nm with microplate reader.

Intracellular internalization profile of nanoparticles in cells. (1) MCF-7 cells and

MCF-10A cells were first cultured in a confocal dish for 24 h. Then, the UCNPs@PAA-$DNA_{1/2}$ (100 μg/mL) in pH=6.5 or 7.4 DMEM culture medium were respectively added to the confocal dish. The cells were incubated for 12 h. After that, fresh DMEM culture medium was added to instead of the original solution. The cells were washed with PBS buffer twice to remove the residual nanoparticles. Then they were observed by confocal laser scanning microscopy (CLSM) and confocal images were acquired. (2) The cells were pre-incubated with 50 μM of free FA for 30 min prior to combining FA receptors.

Flow imaging of cells. MCF-7 and MCF-10A cells were incubated in the presence of UCNPs@PAA-$DNA_{1/2}$ (100 μg/mL) at pH=6.5 or 7.4 for 12 h and the mean fluorescence of 8000 cells were taken by flow cytometry. Similarly, the cells were pre-incubated with 50 μM of free FA for 30 min prior to combining folic acid receptors.

***In vivo* fluorescence imaging.** Two groups of mice bearing MCF-7 tumors were treated with UCNPs@PAA-DNA1(Ce6) or UCNPs@PAA-$DNA_{1/2}$ via intravenous injection, respectively. UCNPs@PAA-DNA1(Ce6) was chosen as control group by modifying Ce6 on the surface of UCNPs@PAA by a ethanediamine linker. At 8 h post-injection, the mice were dissected and fluorescence images were acquired with organs and tumor.

Animal tumor xenograft models.

Nude mice (4-6 week old, female, ~20 g) were fed with normal conditions of 12 h light and dark cycles and given access to food and water ad libitum. In order to establishing the tumor xenograft models, MCF-7 cells were approximately 1×10^6 cells in 150 μL RPMI 1640 and injected subcutaneously into the flanks of the nude mice.

The tumor volume (V) was computed as $V=L\times W^2/2$ by measuring length (L) and width (W). The relative tumor volumes were calculated for each sample as V/V_0 (V_0 was the original tumor volume). The treatments were carried out only once when the tumor volume reach to about 100 mm^3.

***In vivo* antitumor efficacy via intravenous injection.** When the tumor volume reached to approximately 150 mm^3 the tumor-bearing mice were divided into four groups. The treatments were respectively PBS only, laser only, UCNPs@PAA-$DNA_{1/3}$ combined with laser irradiation and UCNPs@PAA-$DNA_{1/2}$ combined with laser irradiation. 100 μL PBS, 100 μL 1.0 mg/mL UCNPs@PAA-$DNA_{1/3}$ or UCNPs@PAA-$DNA_{1/2}$ in PBS were intravenously injected into the mice via tail vein. After 8 h, laser treatment was performed on the tumor region with 980 nm laser at a power of 1.5 $W\cdot cm^{-2}$ for 5min. The tumor size and the body weights of the mice were observed every other day for 14 days (day 0, 2, 4, 6, 8, 10, 12 and 14).

Table S1. DNA sequences used in this work.

	Sequence
DNA1	5'-NH_2-AAAAAAAAAAAA-FA-3'
DNA2	5'-NH_2-CCCTAACCCTAACCCTAACCC-Ce6-3'
DNA3	5'-NH_2-CCATAACTCTAACGCTAACTC-Ce6-3'
DNA4	5'-BHQ1-CCCTAACCCTAACCCTAACCC-Cy3-3'

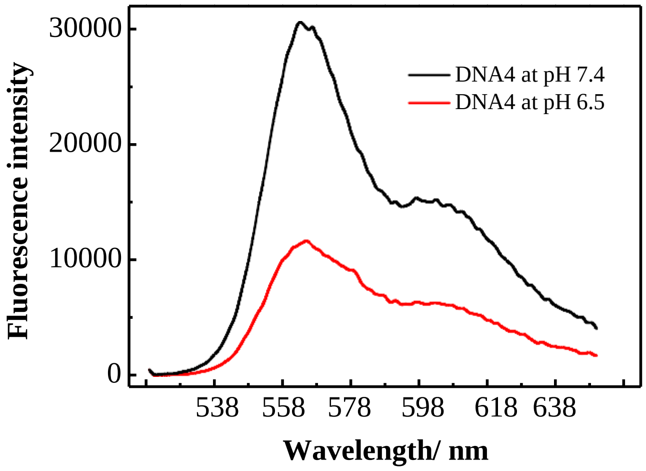

Fig. S1 Fluorescence intensity of DNA4 at pH 7.4 (black) and 6.5 (red).

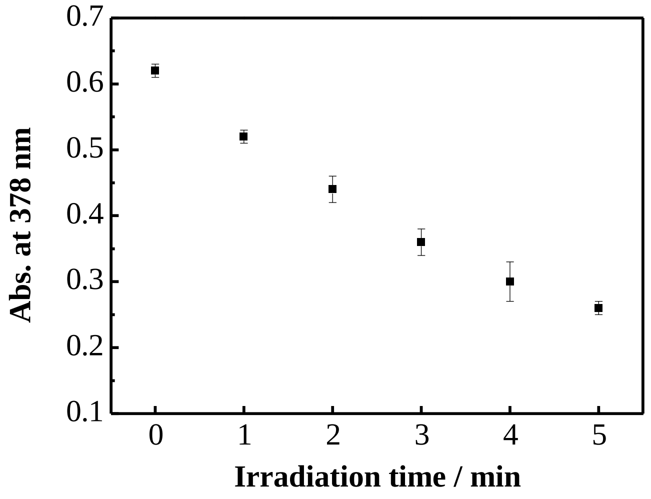

Fig. S2 Kinetic study of UCNPs@PAA-DNA$_{1/2}$ for $^1O_2$ production under the irradiation of 1.5 W·cm^{-2} at pH 6.5.

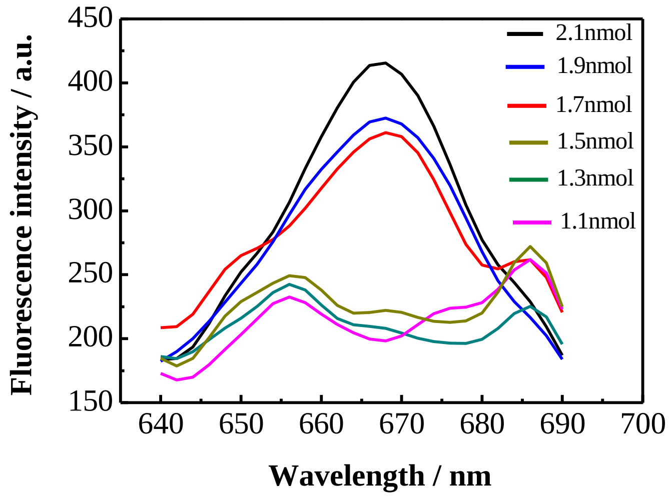

Fig. S3 Fluorescence spectra of the supernate of UCNPs@PAA solution after reaction with different concentration of DNA2.

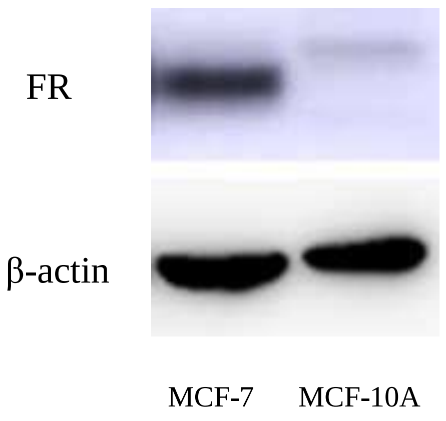

Fig. S4 Western blot of FR expression in MCF-7 cells and MCF-10A cells.

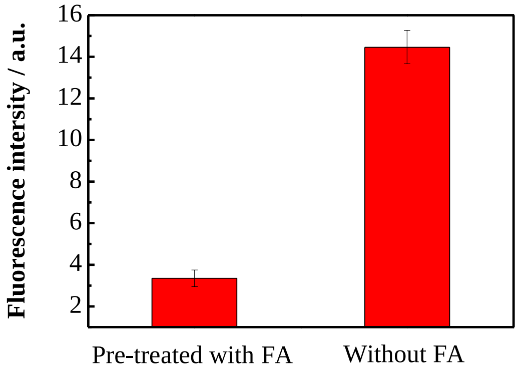

Fig. S5 Mean fluorescence intensity of MCF-7 cells incubated with UCNPs@PAA-DNA_{12} at pH 6.5 when cells were pre-treated with FA or not.

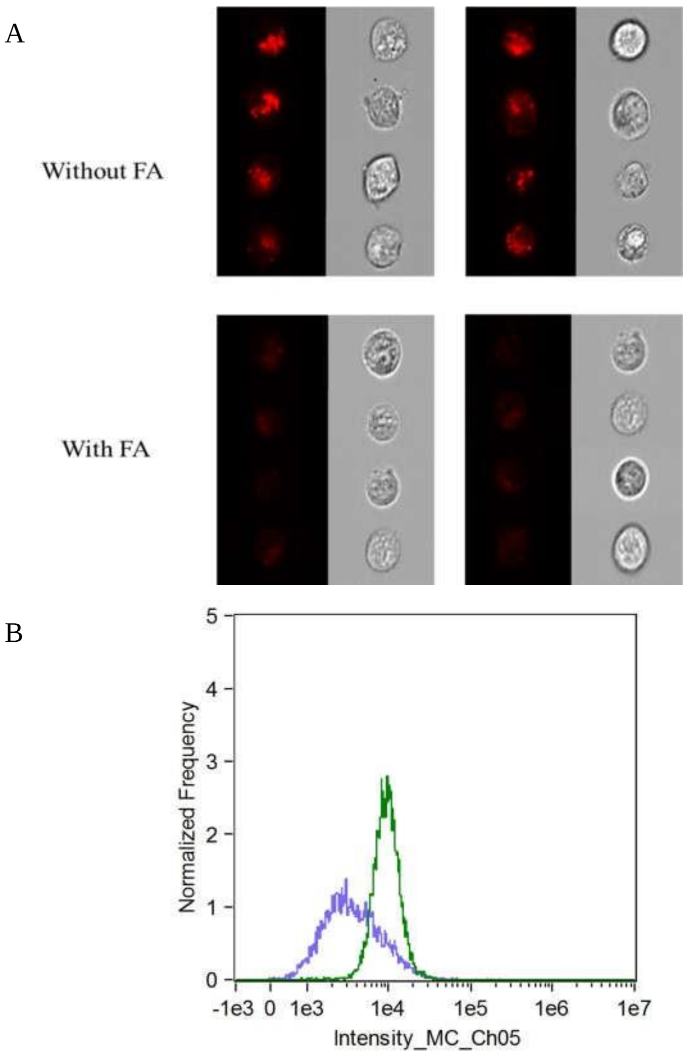

Fig. S6 (A) IFC images of MCF-7 cells incubated with UCNPs@PAA-$DNA_{1/2}$ at pH 6.5 after cells were pre-treated with FA or not. (B) Fluorescence intensity distribution of cells in the two samples.

Table S2. Mean fluorescence intensity of data in Figure S3B.

Sample	Mean fluorescence intensity
With FA	10070.03
Without FA	5008.34

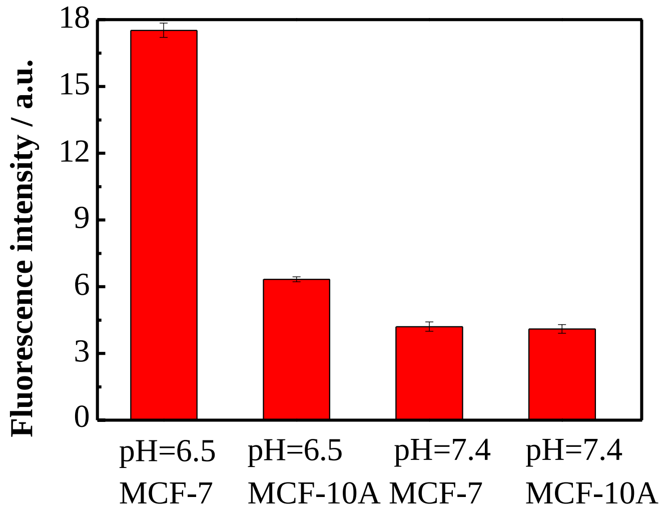

Fig. S7 Fluorescence quantification of MCF-7 and MCF-10 cells after incubated with UCNPs@PAA-$DNA_{1/2}$ at pH 6.5 or 7.4.

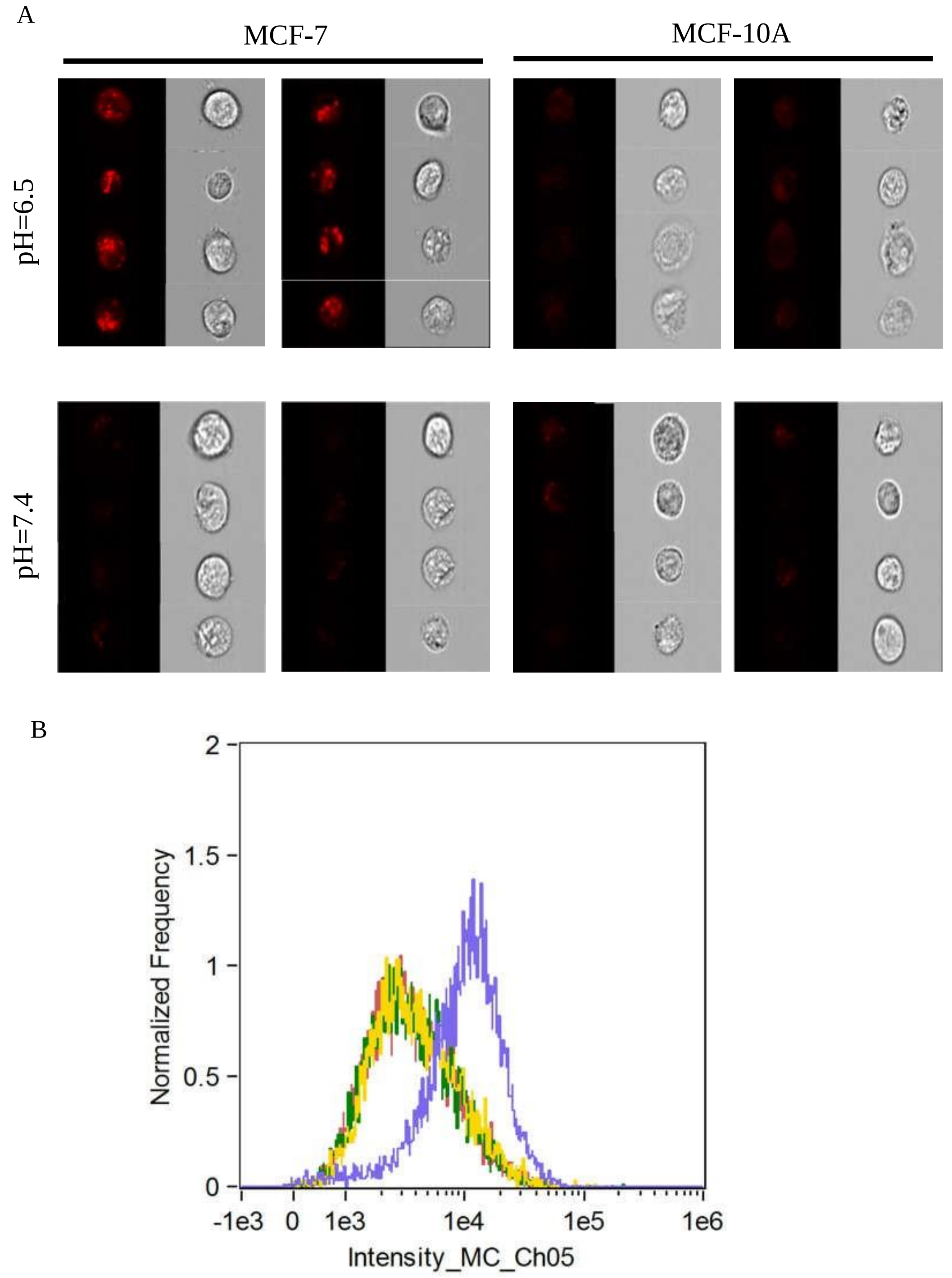

Fig. S8 (A) IFC images of MCF-7 and MCF-10 cells after incubated with UCNPs@PAA-$DNA_{1/2}$ at pH 6.5 or 7.4. (B) Fluorescence distribution of cells in each sample.

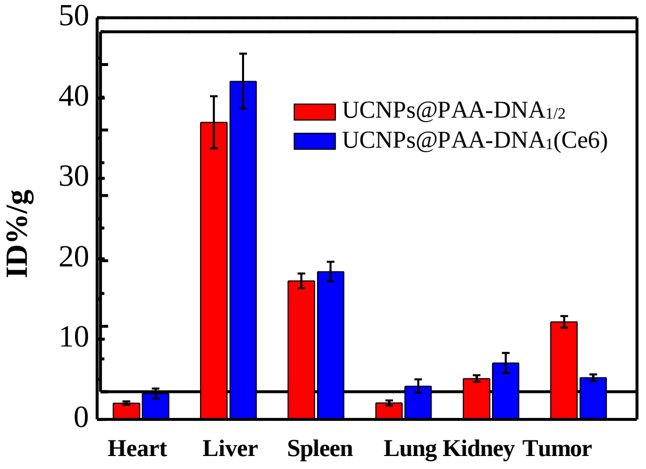

Fig. S9. Biodistribution of UCNPs@PAA-$DNA_{1/2}$ and UCNPs@PAA-DNA_1(Ce6) at 8 h post injection.

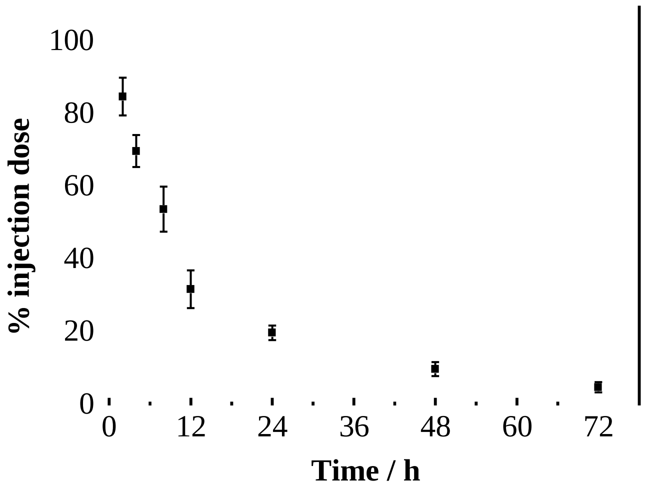

Fig. S10 Retention of UCNPs@PAA-$DNA_{1/2}$ in the mice at different post injection time.

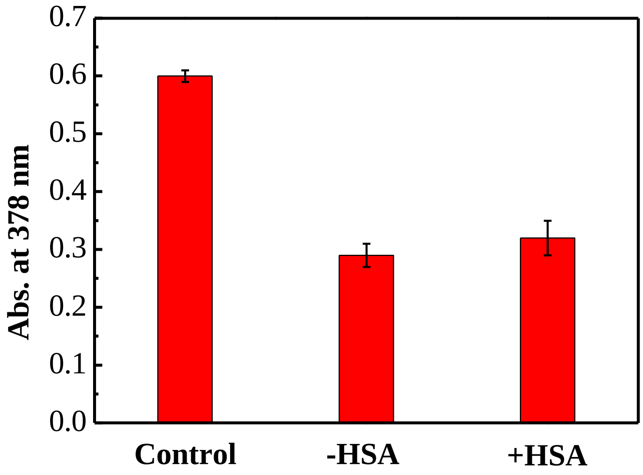

Fig. S11 Adsorption of ABMD at 378 nm when UCNPs@PAA-$DNA_{1/2}$ was irradiated with or without human serum albumin (HAS, 10 mg/mL) at pH 6.5.

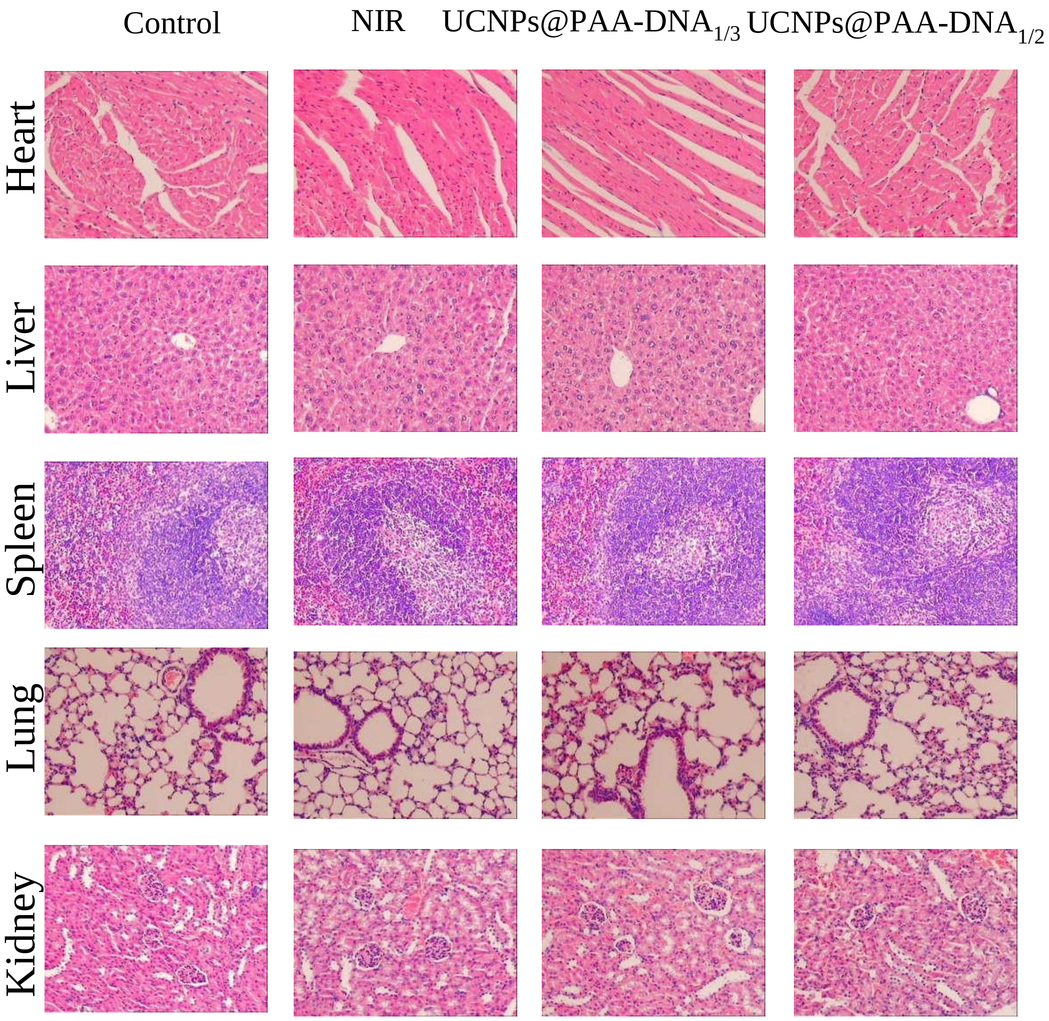

Fig. S12 H&E staining of five major organs in mice received different treatments.
